# Putting organelles in their place

**DOI:** 10.7554/eLife.69422

**Published:** 2021-05-21

**Authors:** Patricia Ulm, Verena Jantsch

**Affiliations:** 1 Department of Chromosome Biology, Max Perutz Laboratories, University of Vienna, Vienna BiocenterViennaAustria

**Keywords:** LINC complexes, nuclear envelope, nesprin, nuclear positioning, ER, cellular organisation, *C. elegans*

## Abstract

Experiments in *C. elegans* reveal new insights into how the ANC-1 protein helps to anchor the nucleus and other organelles in place.

**Related research article** Hao H, Kalra S, Jameson LE, Guerrero LA, Cain NE, Bolivar J, Starr DA. 2021. The Nesprin-1/-2 ortholog ANC-1 regulates organelle positioning in *C. elegans* independently from its KASH or actin-binding domains. *eLife* **10**:e61069. doi: 10.7554/eLife.61069

Cells contain an assortment of organelles which each have their own specialized role. To work correctly, most organelles need to be properly positioned within the cell. For example, mis-localization of the cell’s largest organelle, the nucleus, has been observed in neuromuscular diseases, such as Emery-Dreyfuss muscular dystrophy (Luxton and Starr, 2014).

Current models suggest that positioning of the nucleus relies on a complex called LINC (short for Linker of Nucleoskeleton and Cytoskeleton), which is made up of proteins that contain either a SUN or KASH domain. The SUN proteins (SAD1, UNC-84) sit across the inner nuclear membrane and connect to structures in the nucleus, such as chromatin and the nuclear lamina, and the KASH proteins (Klarsicht, ANC-1, Syne Homology) span across the outer nuclear membrane and interact with proteins in the cytoskeleton. The SUN and KASH domains of these proteins join together to form a bridge that mechanically couples the nucleus and cytoskeleton, which helps to anchor the nucleus in the right place (Kim et al., 2015).

This model of how nuclear positioning works is primarily based on experiments in *Caenorhabditis elegans* worms with mutations in the genes for either the UNC-84 or ANC-1 protein (Starr and Fridolfsson, 2010). The hypodermis of adult wild-type worms is made up of several huge hyp-7 cells (or syncytia) which each contain 139 evenly spaced nuclei (Shemer and Podbilewicz, 2000), making them a useful system for investigating nuclear anchorage. According to the model, if the nuclei in hyp-7 cells are exclusively anchored via the SUN–KASH bridge, then loss of the genes for UNC-84 or ANC-1 should have an identical effect and result in the same amount of nuclear clustering. However, in 2018, a group of researchers made a puzzling discovery: they found that deleting the gene for ANC-1 resulted in more severe nuclear clustering than removing the gene for UNC-84 (Cain et al., 2018). Now, in eLife, Daniel A Starr and co-workers from University California, Davis – including Hongyan Hao as first author – report that the model for how the nucleus is positioned may need re-defining (Hao et al., 2021).

The team (which includes some of the authors involved in the 2018 study) found, as expected, that removing the gene for ANC-1 led to nuclear clustering in hyp-7 cells, indicating that nuclear anchorage had been lost (Figure 1). The untethered nuclei also disrupted the network of microtubules in the cytoskeleton, and appeared much smaller and less rounded, suggesting that the cells lacked mechanical stability. In *C. elegans*, the ANC-1 protein contains multiple domains: an actin-binding domain at its N-terminus, several cytoplasmic domains that likely bind to other proteins, a transmembrane domain, and a KASH domain at its C-terminus (Gundersen and Worman, 2013; Starr and Han, 2002). To gain a better understanding of how ANC-1 positions the nucleus, Hao et al. deleted these different domains, either separately or in combination, to see how this affected the protein’s role in the cell.

**Figure 1.**
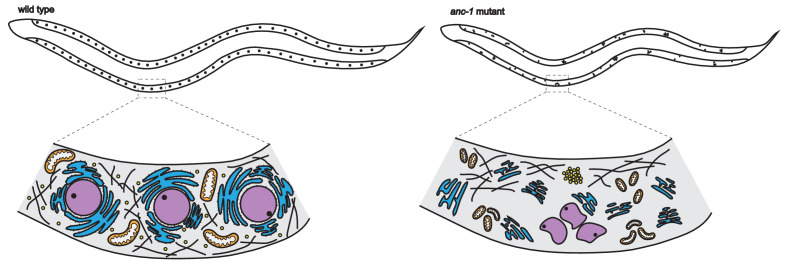
Loss of ANC-1 leads to unanchored and misshaped organelles and a smaller body size in worms. The hypodermis of *C. elegans* worms (top schematic) is made up of hyp-7 cells which contain over a hundred nuclei (represented as black dots). In wild-type worms (left), the KASH protein ANC-1 (depicted as spikes) localizes to the membrane of the nucleus and endoplasmic reticulum (ER). As a result, the nuclei (purple) are spherical and evenly spaced, and the ER (blue), mitochondria (orange) and lipid droplets (yellow) are well anchored. The microtubule network (black lines) is also evenly distributed throughout the cytoplasm. Meanwhile, in mutant worms lacking the gene for ANC-1 (right), the ER and mitochondria are fragmented, and the nuclei are unanchored and clustered together. Lipid droplets are also clustered and the microtubule network is disrupted by the movement of the untethered organelles. This causes the mutant worm to have a smaller body size and the nuclei in its hyp-7 cells to be mispositioned.

Image credit: Patricia Ulm.

Deletion of the KASH domain only caused moderate nuclear clustering, and loss of the actin-binding domain did not generate any nuclear anchorage defects. In contrast, deleting parts of ANC-1 protein that sit within the cytoplasm and likely bind to other cytoskeleton proteins led to more severe positioning defects. Notably, nuclear mispositioning was greatly increased in double mutants lacking both a functional SUN domain in UNC-84 and a cytoplasmic domain of ANC-1. This synergistic effect suggests that nuclear positioning is likely controlled by cooperation between two distinct domains of ANC-1: the cytoplasmic domain and the domain that binds to UNC-84 in the SUN-KASH bridge.

Previous studies have shown that ANC-1 also controls the distribution of mitochondria (Starr and Han, 2002). Therefore, Hao et al. investigated whether ANC-1 is also essential for positioning the mitochondria and another organelle called the endoplasmic reticulum (or ER for short). In mutant worms lacking the gene for ANC-1, they found unanchored fragments of mitochondria and the ER along with clusters of lipid droplets (Figure 1). As seen for the nucleus, deleting the KASH domain only resulted in mild defects in ER positioning, suggesting that localization of the ER also mainly relies on parts of the ANC-1 protein outside the KASH domain.

These findings suggest that ANC-1 positions the nucleus and ER largely independently from the KASH domain. Further experiments revealed that the ANC-1 protein is also located on the membrane of the ER. This led Hao et al. to propose a new cytoplasmic integrity model in which ANC-1 localizes to both the ER and nuclear membranes, and reaches out to correctly position organelles via an interconnecting network that permeates the entire cytoplasm.

This study raises the question of how ANC-1 can position and hold organelles in place with little or no help from its KASH and actin-binding domains. Notably, studies in mice have also shown that the actin-binding domains of Nesprin1 (mouse ANC-1) are not essential to anchor the nucleus within the cytoplasm (Stroud et al., 2017). Therefore, some important questions remain: which cytoskeletal component(s) work with ANC-1 to correctly position organelles and confer mechanical stability to the cell? Are the neuromuscular diseases associated with mutations in the LINC complex the result of alterations in cytoplasmic integrity rather than nuclear anchorage defects? Further experiments using *C. elegans* as a model system may help to answer these questions and shed further light on how ANC-1 anchors organelles in place.
